# Meaning in life and health care use: findings from a nationally representative study of older adults in Germany

**DOI:** 10.1186/s12877-019-1389-3

**Published:** 2019-12-23

**Authors:** André Hajek, Hans-Helmut König

**Affiliations:** Department of Health Economics and Health Services Research, University Medical Center Hamburg-Eppendorf, Hamburg, Hamburg Germany

**Keywords:** Health care utilization, Health services needs and demand, Purpose in life, Meaning in life, Sense of life

## Abstract

**Background:**

There is a lack of studies examining the link between meaning in life and health care use. Meaning in life refers to a sense of comprehension and significance in life. Consequently, the purpose of this study was to investigate the association between meaning in life and health care use.

**Methods:**

Cross-sectional data from the German Ageing Survey, a nationally representative sample of older adults, was used for this study (in the analytical sample: *n* = 3850; year 2002). The frequency of GP and specialist visits in the past 12 months were used as outcome measures. Meaning in life was assessed using a single item measure. Based on Andersen’s model, covariates were selected. Sex, age, family status, occupational status, income, self-rated health, physical functioning, depressive symptoms, and the number of physical illnesses were adjusted for in a multiple regression analysis.

**Results:**

After adjusting for various potential confounders, there was a positive association between meaning in life and GP (IRR: 1.04, 95%-CI: 1.01–1.08) as well as specialist visits (IRR: 1.07, 95%-CI: 1.02–1.12) in a multiple regression analysis. With the exception of employment status (retired vs. employed), income and need factors, no covariates were consistently associated with both outcome measures.

**Conclusions:**

This study highlighted the association between meaning in life and health care use. Our results indicate that there are other factors that are associated with health care use, beyond need-variables. This might help to identify individuals at risk for under- or overuse of health care services.

## Background

In order to be able to manage health care use, it is important to identify its determinants. Drawing on Andersen’s behavioural model, there are numerous studies that have analysed the correlates of health care use (HCU). This model distinguishes between predisposing characteristics such as gender or age, enabling factors (e.g., income) and need factors (e.g., chronic illnesses or self-rated health). A systematic review concluded that need factors in particular shape HCU [1]. This is also important because need factors increases with age. Demographic ageing already places a considerable burden on health care systems [2, 3].

However, some recent studies have demonstrated that other factors exist shown to be important for individuals’ HCU, even after adjusting for the factors mentioned in Andersen’s behavioral model. For example, it has been demonstrated that psychological factors, personality characteristics or general locus of control are also important [4–9]. However, meaning in life has largely been unexplored in HCU research. According to Reker [10] meaning in life can be defined as “having a sense of direction, a sense of order and a reason for existence, a clear sense of personal identity, and a greater social consciousness” (p. 710). A landmark study by Kim et al. [11], for example, showed that purpose in life is associated with the use of several preventive health care services. However, as already argued by George and Park, meaning and purpose are two separate, but related, constructs [12]. For example, they differ in their correlates [12]. While meaning in life refers to a sense of comprehension and significance in life, purpose in life refers to a sense of goals, aims, as well as direction in life [12]. Furthermore, it is worth noting that the study by Kim et al. [11] focused on *preventive* health care services, whereas this study focuses on General Practitioner (GP) and specialist visits in general.

Hence, the purpose of this study was to investigate the association between meaning in life and HCU based on a large population-based sample of individuals ≥40 years. Investigating individuals aged 40 and above is particularly relevant because need factors increase with age. With regard to possible mechanisms, when individuals do not perceive their life as meaningful, it is plausible that they would not invest in their health and therefore might report infrequent outpatient physician visits (e.g., underuse of preventive health care services). Knowledge about this association might help to address individuals at risk for infrequent doctor visits. This is important because randomized controlled trials have demonstrated that meaning in life can be modified [13, 14].

With respect to the health care system in Germany, it is worth noting that health insurance is compulsory. Approximately 90% of the population are enrolled in the statutory health insurance (SHI); the remaining 10% are insured under the private health insurance (PHI). Self-employed individuals, civil servants and individuals who exceed a certain income-threshold can choose between SHI and PHI. Both types provide comprehensive health care for their members. Outpatient specialists can be consulted without a GP referral. For further details with regard to the health care system in Germany, please see Busse et al. [15].

## Methods

### Sample

The current study used data from the German Ageing Survey (DEAS), which started in 1996 (first wave). For reasons of data availability, our study was restricted to the second wave, which took place in 2002, as our key independent variable was only assessed in the second wave.

The Federal Ministry for Family Affairs, Senior Citizens, Women and Youth funded the DEAS study. The DEAS study is a representative study of individuals residing in private households (40 years and over). Therefore, the main inclusion criterion was that individuals had to be at least 40 years old. More specifically, first time participants were included when they met the following criteria: [1] born between 1929 and 1974, [2] living in a private household (which means that individuals residing in institutionalized settings were excluded). For panel participants, inclusion criteria were: [1] one or more valid interviews in former waves, [2] willingness to participate in the panel (written consent given by baseline participants), [3] still alive and not living abroad. Various topics (e.g., perception of ageing, social support, health, occupational status, retirement) are covered in the DEAS study.

In the first wave, 4838 individuals were interviewed (50% response rate) and 5194 individuals took part in the second wave (38% response rate). In the first wave, the gross sample included 9613 individuals. Thereof, 3268 individuals (34.0%) refused participation. Other minor reasons for non-response were, for example, that individuals were temporary ill (108 individuals, 1.1%) or permanently ill (383 individuals, 4.0%). Neller showed that the response rate reported in the DEAS study is similar compared with other large survey studies that have taken place in Germany [16]. Klaus et al. provided additional details with regard to the DEAS study [17]. In our analytical sample (i.e., individuals included in regression analysis), *n* = 3850 individuals were included (with number of specialist visits as outcome measure; *n* = 3844 with number of GP visits as outcome measure).

Written informed consent was provided by all individuals. An ethical statement for the DEAS study was not needed, as the criteria for it were not met (e.g., examination of patients, risk for the respondents, or the use of invasive methods).

### Dependent variable

The use of outpatient physician visits (first dependent variable: number of GP; second dependent variable: number of specialist visits) in the preceding 12 months was assessed in the DEAS study. Several medical specialties were reported in the DEAS study. The number of GP and specialist visits was quantified as: never; once; 2–3 times; 4–6 times; 7–12 times; more often. Following Bock et al. and Flennert et al. [4, 18], it was recoded as: “never” = 0; “once” = 1; “2–3 times” = 2.5; “4–6 times” = 5; “7–12 times” = 9.5; and “more often” = 13.

### Independent variables

A single item (based on WHOQOL-BREF [19]) with clear face validity was used to measure meaning in life: “To what extent do you feel your life to be meaningful?” [1 = not at all; 2 = a little; 3 = a moderate amount; 4 = very much; 5 = extremely]. A recent study has provided evidence for the reliability and validity of this single item measure [20].

Based on Andersen’s behavioral model [21], covariates were selected. Namely, predisposing characteristics such as sex, age, marital status (married, and living together with spouse; married, and living separated from spouse; widowed; divorced; single), and occupational status (employed; retired; other: not employed) were controlled for. With regard to enabling factors, income (household net equivalent income) was adjusted for.

With regard to need variables, self-rated health (from 1 = very good to 5 = very bad), physical functioning (subscale physical functioning of the SF-36 [22]; ranging from 0 = worst to 100 = best), depressive symptoms (15 item version of the Center for Epidemiological Studies Depression Scale [23], from 0 to 45, higher values correspond to more depressive symptoms), and the number of chronic illnesses such as diabetes or cancer (ranging from 0 to 11) were adjusted for.

In a sensitivity analysis, other factors that may affect the link between meaning in life and HCU were adjusted for, i.e. religious affiliation (protestant church (not including free churches), roman catholic church, another Christian community, another non-Christian community, no religious group), health locus of control (from 1 = I have practically no influence on my health to 4 = I have strong influence on my health) and network size (number of important people in regular contact, ranging from 0 to 9). In further sensitivity analysis, the continuous outcome measures were replaced by categorical outcome measures (please see the section dependent variables for categories).

### Statistical analysis

First, sample characteristics were displayed. Subsequently, negative binomial regression analysis were conducted with GP and specialist visits as outcome measures, respectively [24]. Due to the nature of the data (count data; distribution of visits was positively skewed), we used this type of regression analysis [24–26]. Further details with regard to negative binomial regressions are given by Hardin et al. [24]. Meaning in life was our key independent variable. Several potential confounders were adjusted for. The criterion for statistical significance was set at *p* < .05. Analyses were performed using Stata 15.1 (StataCorp, College Station, Texas, USA).

## Results

### Sample characteristics

Sample characteristics are depicted in Table 1. In total, 51.5% of the sample were male and the mean age was 61.0 years (±12.0 years). Average meaning of life was 4.1 (±0.9; Variance: .79; Skewness: − 1.19; Kurtosis: 4.79), average GP visits were 4.2 (±3.7) and average specialist visits were 3.4 (±4.0). Further details are provided in Table 1.

**Table 1:** Sample characteristics (wave 2; *n* = 4655)

Variables	N (%) / Mean (SD)
Sex: - Male: N (%)	2397 (51.5%)
– Female	2258 (48.5%)
Age in years: Mean (SD)	61.0 (12.0)
Family status: - Married, living together with spouse: N (%)	3364 (72.3%)
– Married, living separated from spouse	91 (2.0%)
– Divorced	377 (8.1%)
– Widowed	579 (12.4%)
– Single	242 (5.2%)
Employment status: - Employed: N (%)	1744 (37.5%)
– Retired	2159 (46.4%)
– Other: not employed	752 (16.1%)
Income (in Euro): Mean (SD)	1455 (813)
Self-rated health (from 1 = very good to 5 = very bad): Mean (SD)	2.5 (0.9)
Depressive symptoms: Mean (SD)	7.5 (6.6)
Physical functioning: Mean (SD)	83.5 (23.6)
Number of physical illnesses: Mean (SD)	2.3 (1.9)
Meaning in life (from 1 = not at all to 5 = extremely): Mean (SD)	4.1 (0.9)
GP visits in the past 12 months: Mean (SD)	4.2 (3.7)
GP visits in the past 12 months: N (%)	
“Never”	600 (12.9%)
“Once”	749 (16.1%)
“2–3 times”	1249 (26.8%)
“4–6 times”	1016 (21.8%)
“7–12 times”	782 (16.8%)
“more often”	259 (5.6%)
Specialist visits in the past 12 months: Mean (SD)	3.4 (4.0)

Notes: N = number; SD = standard deviation

### Regression analysis

Findings of regression analysis are shown in Table 2. The parameter estimates were converted to incidence rate ratios (IRRs) by exponentiation (for ease of interpretation). IRRs can be interpreted as the percent change in the outcome variable (GP or specialist visits) associated with a one-unit change in the independent variable (holding all other variables constant). The presence of multicollinearity was investigated based on the variance inflation criterion. The largest variance found was 3.13, which shows that multicollinearity was not present. In the analytical sample, *n* = 3850 individuals were included (with number of specialist visits as outcome measure; *n* = 3844 with number of GP visits as outcome measure).

**Table 2:** Determinants of GP as well as specialist visits. Results of negative binomial regressions

Independent variables	GP visits	Specialist visits
Meaning in life (from 1 = not at all to 5 = extremely)	1.04**	1.07**
	(1.01–1.08)	(1.02–1.12)
Female (Ref.: male)	1.05+	1.78***
	(1.00–1.11)	(1.66–1.91)
Age	1.00+	0.99*
	(1.00–1.01)	(0.99–1.00)
Family status: - Married, living separated from spouse (Ref.: Married, living together with spouse)	0.94	1.34*
	(0.77–1.16)	(1.06–1.70)
– Divorced	0.91+	1.03
	(0.83–1.01)	(0.91–1.16)
– Widowed	1.02	0.90+
	(0.94–1.10)	(0.80–1.01)
– Single	0.94	0.94
	(0.82–1.07)	(0.79–1.11)
Employment status: - Retired (Reference: Employed)	1.22***	1.22**
	(1.11–1.34)	(1.08–1.38)
– Other: not employed	1.05	1.15**
	(0.96–1.15)	(1.04–1.29)
Log income	0.88***	1.17***
	(0.83–0.93)	(1.08–1.27)
Self-rated health (from 1 = very good to 5 = very bad)	1.27***	1.24***
	(1.23–1.33)	(1.18–1.31)
Depressive symptoms (from 0 (no depressive symptoms) to 45 (severe depressive symptoms))	1.00*	1.01***
	(1.00–1.01)	(1.01–1.02)
Physical functioning (from 0 (worst) to 100 (best))	1.00***	1.00*
	(1.00–1.00)	(1.00–1.00)
Number of physical illnesses (from 0 to 11)	1.09***	1.14***
	(1.08–1.11)	(1.11–1.16)
Constant	3.17***	0.20***
	(1.87–5.38)	(0.09–0.41)
Observations	3844	3850
Pseudo R^2^	.056	.045

Incidence rate ratios were reported; 95% CI in parentheses; *** *p* < 0.001, ** *p* < 0.01, * *p* < 0.05, + *p* < 0.10

Adjusting for numerous potential confounders, multiple negative binomial regressions revealed that meaning in life was associated with GP (IRR: 1.04, 95%-CI: 1.01–1.08) as well as specialist visits (IRR: 1.07, 95%-CI: 1.02–1.12). With the exceptions of employment status (retired vs. employed), income and need factors (including self-rated health, depressive symptoms, physical functioning and number of physical illnesses), none of the covariates were consistently associated with both outcome measures.

We also tested for non-linearity by including (i) quadratic as well as (ii) cubic terms for meaning in life. However, they did not achieve statistical significance. Moreover, a sensitivity analysis was performed to test the robustness of our findings. Specifically, the main model was extended by adding religious affiliation, health locus of control and network size. However, the association between meaning in life and the outcome measures remained virtually the same (results not shown, but available upon request). Furthermore, we replaced the continuous outcome measures with categorical outcome measures (ordered probit regressions were used). However, in terms of significance, the association between meaning in life and the outcome measures remained almost the same (with GP visits as outcome measure: β = .09, *p* < .001; with specialist visits as outcome measure: β = .09, *p* < .001).

## Discussion

### Main findings

Using data from a nationally representative sample of older adults, the aim of this study was to investigate the association between meaning in life and HCU. After adjusting for various potential confounders (e.g., socioeconomic variables, physical functioning, depressive symptoms, number of physical illnesses and self-rated health), a positive association between meaning in life and GP as well as specialist visits was found in multiple regression analysis.

### Previous research and possible explanations

There is a lack of studies to date that have examined the link between meaning in life and HCU in general. A few studies conducted by Kim and colleagues have investigated the link between use of preventive health care services and aging satisfaction, as well as life satisfaction, among older adults [27, 28]. These studies showed that individuals scoring higher in aging satisfaction tend to use several preventive health care services (e.g., use for cholesterol tests, obtaining a mammogram/x-ray in women or obtaining a prostate exam in men) more often. Similar findings were made for the association between life satisfaction and the use of preventive health care services.

Furthermore, based on data from the Health and Retirement Study (*n* = 7168), another recent study conducted by Kim et al. [11] showed that purpose in life was positively associated with the use of different preventive health care services (such as mammogram/X-ray, pap smear or prostate examination) after adjusting for factors such as age, marital status, or an index of major chronic illnesses. This indicates that purpose in life is linked to health promoting behavior (in that individuals who score high in purpose in life may be more proactive in taking care of their health [11]) Therefore, it appears plausible to us that meaning in life, a closely related construct, was associated with HCU *in general* in our study (even after adjusting for several potential confounders).

Another way to explain our findings may be that individuals in the second half of life who score low in meaning in life might have a lack of drive, decreased health [29] or may feel that they do not belong to the society [30]. Therefore, these individuals may not see the value in visiting the doctor to maintain their health [31]. However, future research is required to elucidate the mechanisms by which meaning in life can affect HCU. The findings of our study provide first evidence on the relationship between meaning in life and HCU in general.

With regard to control variables, (e.g., need-factors like self-rated health) our findings are mostly in line with previous literature. For example, a systematic review conducted by Babitsch et al. [1] showed that increased need-factors in particular are associated with increased HCU.

It is interesting that, for example, worse self-rated health is associated with *increased* HCU in our study, whereas a low meaning in life is associated with *decreased* HCU. We assume that the aforementioned factors (lack of energy, social isolation) may drive the link between meaning in life and HCU. At present, however, there is no evidence to support our assertion.

### Strengths and limitations

This is the first study investigating the association between meaning in life and HCU based on a large nationally representative sample of older adults in Germany. Based on Andersen’s theoretical model, various covariates were included. There is some sample selectivity in the DEAS study. For example, participation rates are lower among, for example, among women, middle-aged (40 to 54 years) and individuals from 70 to 85 years. Further details are given by Klaus et al. [17]. However, this sample selection bias was not found to be important in the DEAS study [17]. A single item was used to measure meaning in life. The underlying reason for physician visits (e.g., preventive or curative) remains unclear. As far as data are available, future studies should investigate whether the link between meaning in life and HCU varies by the reason for consultation. Moreover, data from the second wave of the DEAS study (taking place in 2002) were used. Thus, we cannot dismiss the possibility that the link between meaning in life and HCU differs between past and present cohorts. On the one hand, there might be cohort effects with respect to the meaning of life (“Zeitgeist”). The accessibility of physicians might also have changed (e.g. in terms of waiting times).

Future studies are required to validate our findings based on instruments that are more sophisticated (e.g., meaning in life questionnaire [32]). Furthermore, longitudinal studies are required to establish the long-term role of meaning in life on HCU.

## Conclusions

This study highlighted the association between meaning in life and HCU. Thus, our results indicate that beyond need-variables other factors exist that are associated with HCU. This might help to identify individuals at risk for under- or overuse of health care services.

## Data Availability

The data used in this study are third-party data. The anonymized data sets of the DEAS (1996, 2002, 2008, 2011, 2014, and 2017) are available for secondary analysis. The data has been made available to scientists at universities and research institutes exclusively for scientific purposes. The use of data is subject to written data protection agreements. Microdata of the German Ageing Survey (DEAS) is available free of charge to scientific researchers for non-profitable purposes. The FDZ-DZA provides access and support to scholars interested in using DEAS for their research. However, for reasons of data protection, signing a data distribution contract is required before data can be obtained. Please see for further information (data distribution contract): https://www.dza.de/en/fdz/german-ageing-survey/access-to-deas-data.html

## Abbreviations

DEAS: German Ageing Survey
GP: General Practitioner
HCU: Health care use
IRR: Incidence rate ratio
PHI: Private health insurance
SF-36: Short Form 36
SHI: Statutory health insurance
WHOQOL-BREF: World Health Organization Quality of Life Brief Version
